# Clinical characteristics of two patients with neuronal intranuclear inclusion disease and literature review

**DOI:** 10.3389/fnins.2022.1056261

**Published:** 2022-12-05

**Authors:** Bo Zhao, Miao Yang, Zhiwei Wang, Qiqiong Yang, Yimo Zhang, Xiaokun Qi, Shuyi Pan, Yingxin Yu

**Affiliations:** ^1^Department of Neurology, The First Medical Center, Chinese PLA General Hospital, Beijing, China; ^2^The Second School of Clinical Medicine, Southern Medical University, Guangzhou, China

**Keywords:** neuronal intranuclear inclusion disease, *NOTCH2NLC*, GGC repeat expansion, neurodegenerative disease, PolyG

## Abstract

**Background:**

Neuronal intranuclear inclusion disease (NIID) is a rare chronic progressive neurodegenerative disease, with complex and diverse clinical manifestations and pathological eosinophilic hyaline intranuclear inclusions in the central and peripheral nervous systems and visceral organs. Improvements in diagnostic methods such as skin biopsy and gene testing are helpful in revealing the clinical and genetic characters of NIID.

**Materials and methods:**

We presented two cases of NIID diagnosed by using *NOTCH2NLC* gene testing and skin biopsy. Diffusion weighted imaging (DWI) showed high linear intensity in corticomedullary junction. We also reviewed all the published NIID cases with positive *NOTCH2NLC* GGC repeat expansion and skin biopsy results in PubMed.

**Results:**

Patient 1 was a 63-year-old male who carried 148 GGC repeats and presented with progressive tremor and limb weakness. Patient 2 was a 62-year-old woman who carried 131 GGC repeats and presented with tremors, memory loss and headaches. The most common clinical manifestation of 63 NIID patients in this study was cognitive impairment, followed by tremors. In our study, almost all the patients were from East Asia, the male to female ratio was 1:1.26, with an age of onset of 54.12 ± 14.12 years, and an age of diagnosis of 60.03 ± 12.21 years. Symmetrical high signal intensity at the corticomedullary junction on DWI were revealed in 80.96% of the patients. For the GGC repeat numbers, the majority of GGC repeats were in the 80–119 intervals, with few GGC repeats above 160. The number of GGC repetitions was significantly higher in patients presented with muscle weakness than in other clinical manifestations.

**Conclusion:**

NIID is a neurodegenerative disease caused by aberrant polyglycine (polyG) protein aggregation. NIID mostly occurs in the elderly population in East Asia, with cognitive dysfunction as the most common symptom. Staging NIID based on clinical presentation is inappropriate because most patients with NIID have overlapping symptoms. In our study, there was no significant correlation between the number of GGC repeats and different phenotypes except for muscle weakness. Abnormal trinucleotides repeat and PolyG protein aggregation maybe common pathogenic mechanism in neurodegenerative diseases and cerebrovascular diseases, which needs to be confirmed by more studies.

## Introduction

Neuronal intranuclear inclusion disease (NIID) is a rare chronic progressive neuro degenerative disease, with complex and diverse clinical manifestations and pathological eosinophilic hyaline intranuclear inclusions in the central and peripheral nervous systems, and multiple visceral organs. Before skin biopsy was used to diagnose NIID in 2011, less than 40 cases of NIID had been reported (Sone et al., 2011). With the characteristic findings of pathological features of skin biopsy (Sone et al., 2011), symmetrical high signal intensity in corticomedullary junctions on diffusion weighted imaging (DWI) (Sone et al., 2014), and the genetic GGC repeat expansion in *NOTCH2NLC* gene (Deng et al., 2019; Sone et al., 2019; Tian et al., 2019), more sporadic and familial cases of NIID have been reported. Its clinical spectrum has also been extended to a wide range of neurological disorders and other systemic disorders. Dementia, muscle weakness, leukoencephalopathy, and essential tremor are common clinical manifestations in patients with NIID, some patients can also present with autonomic dysfunction, peripheral neuropathy, ataxia, unconsciousness, sensory disturbance, headache, seizure, oromandibular dystonia, abnormal behavior, peripheral neuropathy, and other neurological damage manifestations (Sone et al., 2005, 2016; Nakamura et al., 2018; Cao et al., 2021). In patients with NIID, other system involvement, such as urinary dysfunction (Nakamura et al., 2018), retinopathy (Nakamura et al., 2020), recurrent vomiting (Cao et al., 2021), miosis, gastrointestinal dysfunction, orthostatic hypotension, arrhythmia, and sexual dysfunction (Lu and Hong, 2021), can also be observed.

According to the age of onset, NIID can be divided into three subgroups: infant form, juvenile form, and adult form (Takahashi-Fujigasaki, 2003). Sporadic and familial forms of NIID have also been reported (Sone et al., 2016). Based on the initial and main symptoms, NIID have been divided into dementia-dominant, parkinsonism-dominant type and muscle weakness-dominant phenotypes (Sone et al., 2016; Tian et al., 2019). As the number of reported NIID cases increases, more clinical subtypes are defined, including tremor-dominant subtype (Yang et al., 2022), ALS subtype (Yuan et al., 2020), movement-disorder dominant subtype (Tian et al., 2022), paroxysmal symptom-dominant subtype (Tian et al., 2022), and peripheral neuropathy subtype (Hong et al., 2022).

Despite the gradual increase in the reported cases of NIID, the pathogenesis, and the correlation between NIID genotype and phenotype remain unclear. Currently, there are lack of large sample size clinical studies analyzing the clinical characteristics of patients with NIID. The purpose of this study was to report two typical adult cases with NIID diagnosed with both genetic and pathological studies and to summarize and analyze the clinical, genetic, pathological, and radiological characteristics of all the reported NIID patients diagnosed with the same criteria.

## Materials and methods

### Subjects

We presented two cases of NIID diagnosed by using abnormal *NOTCH2NLC* gene and skin biopsy, with DWI showing high intensity in corticomedullary junction. We also reviewed all the cases of NIID in PubMed database searching by using the terms “neuronal intranuclear inclusion disease.” Case reports and references were obtained and reviewed. The inclusion criteria were as follows: (1) skin biopsy indicating intranuclear inclusions in the nuclei of fibroblasts, fat cells and ductal epithelial cells of sweat glands, (2) *NOTCH2NLC* gene testing showing abnormal repeated GGC premutation, and (3) detailed medical history. Finally, 63 patients met the inclusion criteria (Chen et al., 2020; Dong et al., 2020; Guo et al., 2020; Ishihara et al., 2020; Li et al., 2020; Liang et al., 2020; Ogasawara et al., 2020; Okamura et al., 2020; Wang et al., 2020; Yuan et al., 2020; Zhang et al., 2020, 2022; Cao et al., 2021; Deng et al., 2021; Huang et al., 2021; Kikumoto et al., 2021; Kotani et al., 2021; Pang et al., 2021; Tachi et al., 2021; Zhao et al., 2021; Liao et al., 2022; Yang et al., 2022). The clinical, radiological, pathological, genetic, electrophysiological features, and cerebrospinal fluid (CSF) results were analyzed.

### Study participants

Two NIID-affected case subjects from mainland China were included for this study. Both of them were recruited from our hospital. All NIID-affected case subjects presented with typical high linear signals in corticomedullary junction in DWI and received skin biopsy and *NOTCH2NLC* gene testing to confirm the diagnosis. The Montreal Cognitive Assessment (MoCA) and/or Mini-Mental State Examination (MMSE) evaluations were conducted for screening cognitive impairment. The patients subsequently underwent lumbar puncture, electromyography (EMG), and electroencephalography (EEG). All patients provided their written informed consent to participate in this study. The Local Ethics Committee of The Sixth Medical Center of the PLA General Hospitals approved this study.

### Brain magnetic resonance imaging

All MRI examinations including conventional T_1_-weighted spin echo images (T_1_WI), T_2_-weighted spin echo images (T_2_WI), fluid attenuation inversion recovery (FLAIR) images, and DWI images were performed on a standard clinical 3.0-T MRI scanner.

### Skin biopsy

Under local anesthesia, a 5 mm diameter skin biopsy specimen was obtained from 10 cm above the lateral malleolus of the affected individual. Histochemical samples were fixed in 10% formalin, embedded in paraffin, and sliced into 6 mm-thickness sections. The sections subsequently were stained with Hematoxylin and Eosin (H&E), and anti-p62 antibody immunofluorescence. Samples for electron microscopic were fixed with 2.5% glutaraldehyde, then incubated in 1% osmium tetroxide, followed by dehydrated with graded acetone, and finally embedded with resin. After stained with uranyl acetate and lead citrate, ultrathin sections were viewed with electron microscopy.

### PCR assays and GGC repeat size determination

For the repeat-primed PCR (RP-PCR) assay, a fluorescein labeled gene-specific primer were utilized for identifying the GGC repeat expansion of *NOTCH2NLC* gene. GC-rich PCR (GC-PCR) was performed to evaluate the number of GGC repeats.

## Results

### Case description

#### Case 1

**FIGURE 1:**
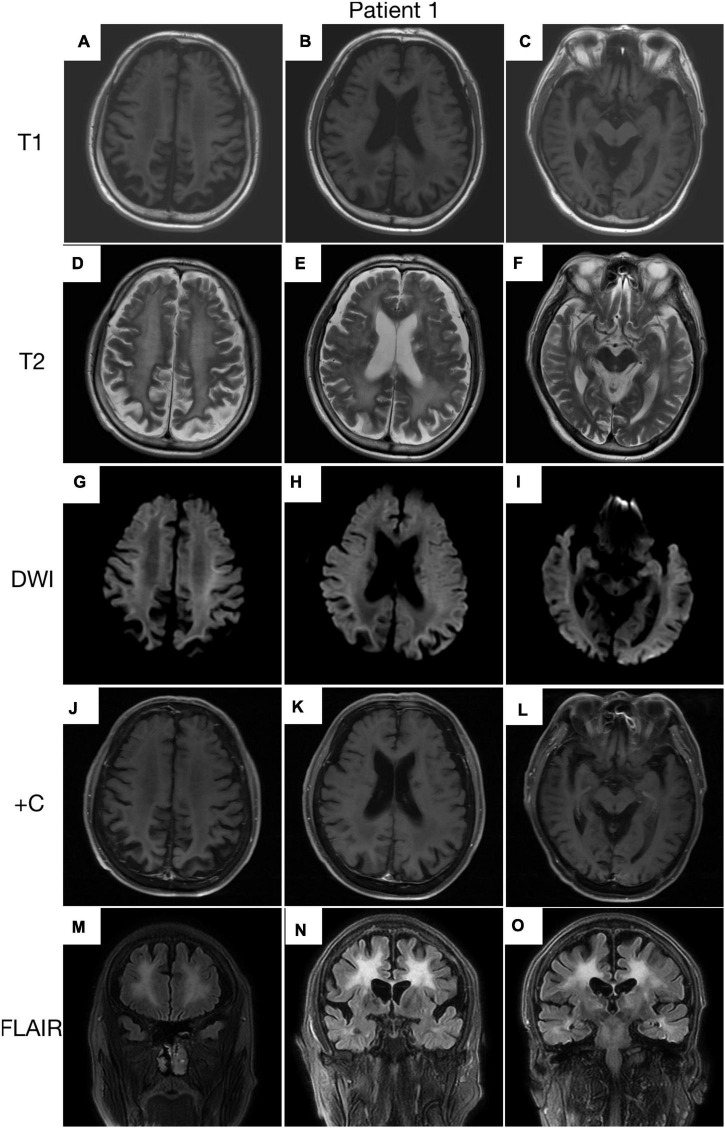
Brain MRI scan of patient 1. Cerebral atrophy was observed on T1 images **(A–C)**. T2 **(D–F)** and FLAIR **(M–O)** images showed severe leukoencephalopathy. Diffusion-weighted imaging (DWI) images revealed high linear signal in the corticomedullary junction **(G–I)**. No significant enhancing lesions were observed **(J–L)**.

**FIGURE 2:**
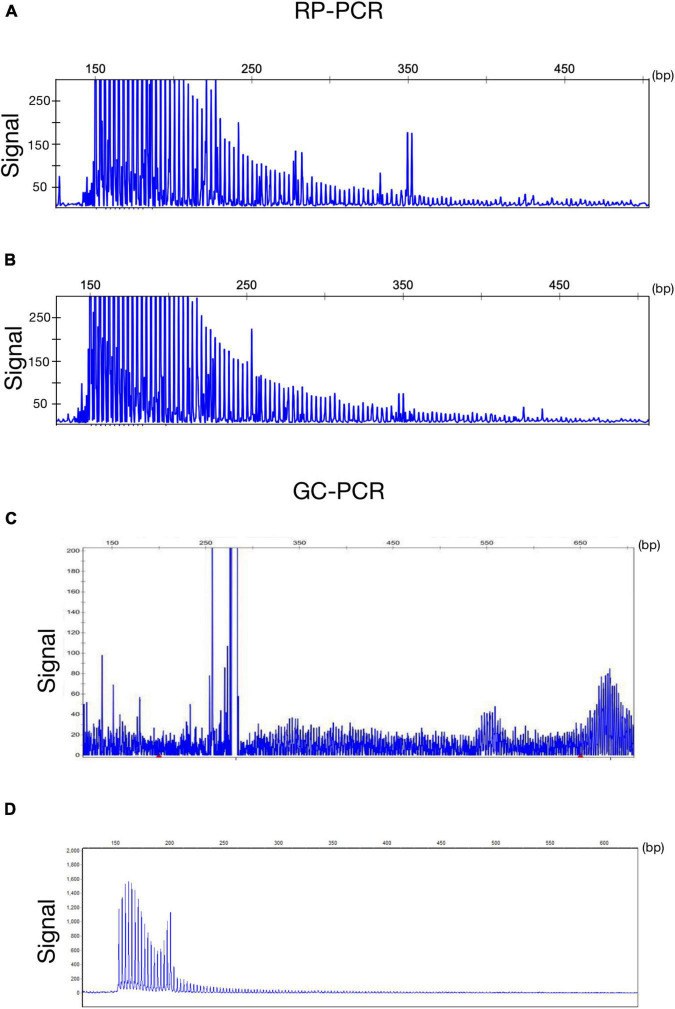
Genetic findings of NIID. Electropherogram of repeat-primed PCR (RP–PCR) analysis shows a saw-tooth tail pattern of the repeat expansion in *NOTCH2NLC*-affected individuals **(A,B)**. Electropherogram of amplicon length PCR (GC–PCR) analysis shows the number of GGC repeats in NIID-affected individuals **(C,D)**.

**FIGURE 3:**
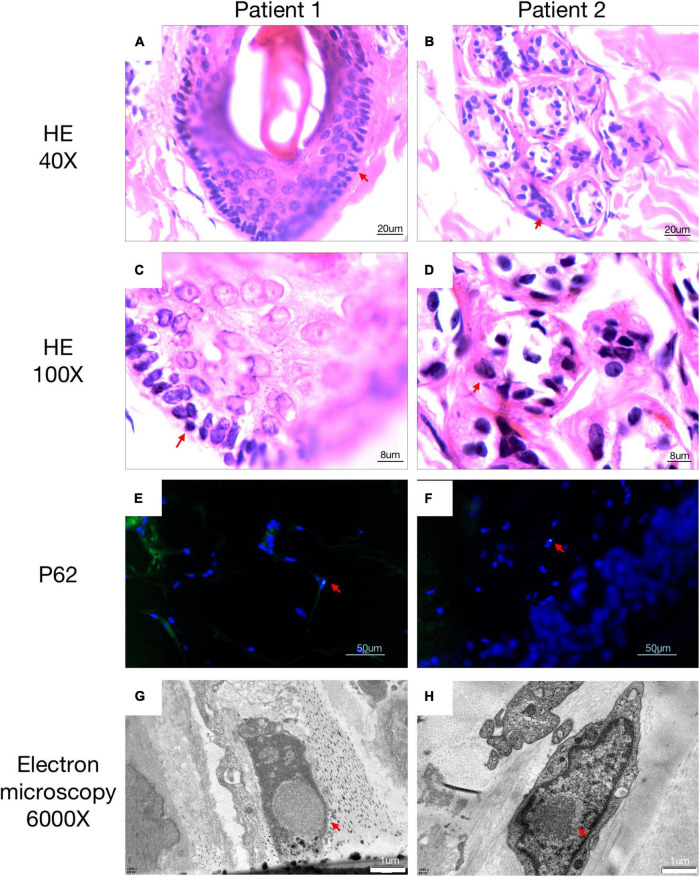
Histopathological findings of NIID. H&E staining of skin biopsy shows the presence of eosinophilic intranuclear inclusion bodies **(A–D)**. Intranuclear inclusions stained with anti-p62 antibody were observed in the 4′,6-diamidino-2-phenylindole di-lactate –positive nuclei by double immunofluorescence staining **(E,F)**. Electron microscopy showed intranuclear inclusions as a pile of round filament material without a limiting membrane **(G,H)**. All positive pathology results were concluded by comparison with a healthy population.

A 63-year-old, right-handed male was admitted to our neurology ward for 10 years of tremor and 6 months of progressive limb weakness. A total of 10 years ago, he began to have intentional tremor of both upper limbs, which was apparent during activities such as eating. The patient was not treated, and the symptoms gradually developed into positional, resting, and intentional tremors in all limbs over the 10 years. And the tremor affected the patient’s ability to walk. Brain MRI-DWI showed corticomedullary junction hyperintensity 6 months ago, he was treated with mecobalamin and vitamin Bs, the above symptoms did not improve significantly. Head CT showed lacunar cerebral infarction in the bilateral basal ganglia region and around parietal ventricles 1 month ago, the patient began to be easily agitated when interacting with others, and occasionally experienced insomnia and loss of recent memory. A year ago, the patient developed blindness in her right eye. The patient had a history of hypertension of more than 10 years and was treated with amlodipine tablets orally. The blood pressure was poorly controlled, with the highest blood pressure reaching 190/100 mmHg and a history of hypertensive retinopathy in the last 1 year. Vitiligo of the extremities has been untreated for more than 60 years. His mother, brother, and niece have similar tremors. The patient’s general condition after hospitalization was good. Physical examination revealed recent memory loss, bilateral visual field deficits, horizontal nystagmus in both eyes when seeing to the right, normal muscle strength and tone in the extremities, decreased tendon reflexes and pharyngeal reflexes bilaterally, and glove and sock hypoesthesia. The MoCA score was 10. Routine blood tests and biochemical tests were unremarkable. Blood tumor markers such as squamous cell carcinoma antigen and CYFRA21-1 were mildly elevated, and concentrations of 8.20 ng/ml (normal: 0∼1.5) and 5.98 ng/ml (normal: 0∼4) respectively. The lumbar puncture pressure, routine CSF analysis and CSF cytology showed no abnormalities. Serum and CSF were negative for oligoclonal band and myelin basic protein. The electrocardiogram (ECG) and EEG were normal. EMG results of peripheral nerve suggested slowed motor and sensory nerve conduction, suggesting nerve myelin sheath damage. No significant abnormalities on ultrasound of the chest and abdominal organs. Both cerebral atrophy and leukoencephalopathy were observed by brain MRI (Figures 1A–F). Brain MRI also revealed high linear intensity signals on DWI along the corticomedullary junction of the bilateral hemisphere, particularly in the parietal, frontal, and occipital lobes, as well as multifocal, punctate regions of low intensity signals on DWI and high signals on T2/FLAIR images involving the lateral paraventricular white matter (Figures 1D–I,M–O). Leukoencephalopathy and cerebral atrophy were also observed. Post-contrast MRI showed no enhancement (Figures 1J–L). We performed skin biopsy and electropherogram of RP-PCR and GC-PCR after the DWI suggested the characteristic lesions. RP-PCR analysis shows a saw-tooth tail pattern (Figure 2A). The results of GC-PCR on *NOTCH2NLC* gene showed that the number of GGC repeat amplifications was 148 (Figure 2C). Immunofluorescence examination and H&E staining of the skin biopsy showed the positive P62 and eosinophilic intranuclear inclusions (Figures 3A,C,E,G). Based on these findings, a diagnosis of NIID was ascertained.

#### Case 2

**FIGURE 4:**
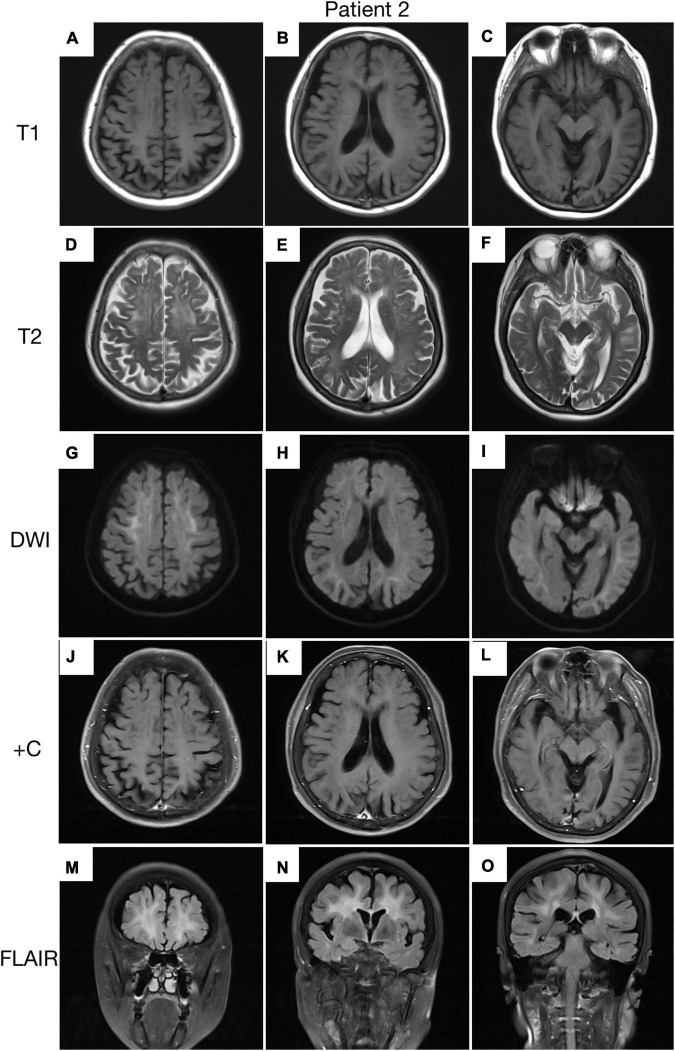
Brain MRI scan of patient 2. Cerebral atrophy was observed on T1 images **(A–C)**. T2 **(D–F)** and FLAIR **(M–O)** images revealed severe leukoencephalopathy. Diffusion-weighted imaging (DWI) images revealed high linear signal in the corticomedullary junction **(G–I)**. No significant enhancing lesions were observed **(J–L)**.

A 62-year-old, right-handed female was admitted to our neurology ward for 6 years of tremor, 1 year of memory loss and 5 months of migraine. The patient developed tremors in both hands 6 years ago, especially when holding objects. She began to frequently forget the position of his own things 1 year ago. She developed migraines without aura 5 months ago. Each week, she had 1 to 2 attacks of mild, unilateral, pulsating pain usually lasting 1–6 h, especially noticeable at night, and occasionally accompanied by nausea, but not by vomiting, photophobia and phonophobia. Over the past 6 months, she had not taken painkillers and headache frequency increased to 3 to 4 times per week, with headaches developing to a moderate level and lasting from half an hour to 8 h with restricted physical activity. The patient had a history of hypertension for more than 10 years, with a maximum blood pressure of 155/95 mmHg, and was treated with long-term oral nifedipine extended-release tablets. The MoCA score was 13, and the MMSE score was 25. The patient had a Bech-Rafaelsen Mania Rating Scale (BRMS) score of 10, suggesting a manic episode. The Geriatric Depression Scale (GDS) score was 14, suggesting mild depression. The Self-Rating Anxiety Scale (SAS) was 38, suggesting moderate anxiety. Apart from the positive pyramidal sign, no significant abnormalities were found on neurological examination. The lumbar puncture pressure, CSF chemical analysis and cytology were normal. Laboratory examinations, routine blood tests and biochemical tests were unremarkable. Serum and CSF were negative for oligoclonal band and myelin basic protein. A total of 24 h ECG suggested inferior wall myocardial ischemia, and the coronary CTA indicated mild stenosis of the right coronary artery. Ultrasonography of chest and abdomen organs showed no obvious abnormality. Peripheral nerve EMG did not show abnormalities. Digital video electroencephalogram revealed some diffuse slow waves, especially in the leads of frontal and temporal regions. Brain MRI-DWI showed corticomedullary junction hyperintensity, especially in the parietal and frontal lobes (Figures 4G–I). Bilateral basal ganglia, lateral ventricle and occipital lobe indicated multiple, patchy low T_1_ and high T_2_/FLAIR signals (Figures 4A–F,M–O), no enhanced lesions were found on contrast MRI (Figures 4J–L). We performed the skin biopsy and found intranuclear inclusion bodies in the sweat gland cells (Figures 3B,D,F,H). A saw-tooth tail pattern was observed by RP-PCR (Figure 2B). A total of 131 GGC repeat expansions in *NOTCH2NLC* gene was found with GC-PCR (Figure 2D). Finally, the diagnosis of NIID was confirmed.

### Literature review

**FIGURE 5:**
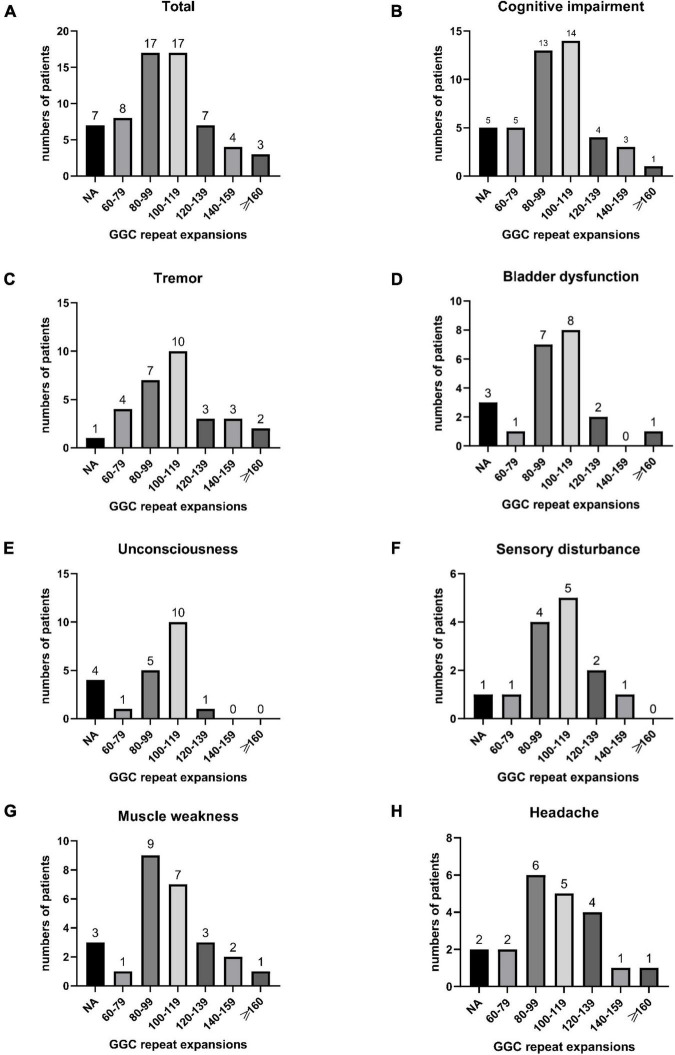
Number of all NIID patients in different GGC repeat number intervals **(A)**; The number of patients with cognitive impairment **(B)**, tremor **(C)**, bladder dysfunction **(D)**, unconsciousness **(E)**, sensory disturbance **(F)**, muscle weakness **(G)**, and headache **(H)** distributed in different GGC repeat number intervals.

**FIGURE 6:**
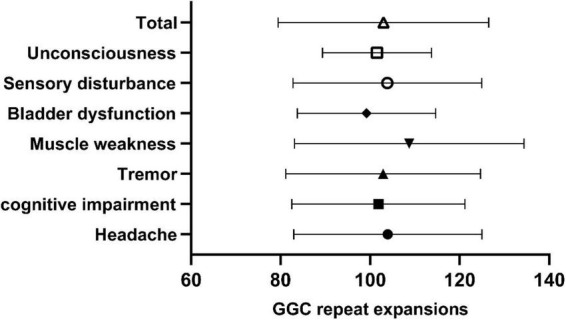
Number of GGC repetitions in NIID patients with different symptoms. Total: 102.98 ± 23.52; Unconsciousness: 101.53 ± 12.17; Sensory impairment: 103.84 ± 21.09; Bladder dysfunction: 99.17 ± 15.46; Muscle weakness: 108.74 ± 25.66; Tremor: 102.885 ± 21.76; Cognitive impairment: 101.84 ± 19.35; Headache: 103.94 ± 21.03.

In our study, the clinical characteristics of 63 patients with NIID, including the 2 cases we reported, were summarized (Table 1). Patients with NIID usually presented in adulthood, with a mean age of onset of 54.12 ± 14.12 years and a mean age of diagnosis of 60.03 ± 12.21 years. The most common initial symptoms are cognitive impairment and tremor, in addition to weakness, dizziness, impaired consciousness, and headache. The most common clinical manifestation in the 63 patients was cognitive impairment (45/56, 80.36%), with a MOCA score of 17.19 ± 6.45 (*n* = 16) and an MMSE score of 20.54 ± 8.85 (*n* = 26). Tremor (30/47, 63.83%) and other Parkinsonian symptoms are not uncommon. Bladder dysfunction is the most common autonomic dysfunction symptom. Some patients also showed signs of peripheral nerve damage such as muscle weakness and sensory deficits, and EMG suggested damage to motor and sensory nerves. A total of 21 patients presented with other neurological deficits such as impaired consciousness, mainly due to encephalitis attacks. Fifty-two of the 63 patients showed high signal in the corticomedullary junction region on DWI. In addition, cerebral white matter lesions and cerebral atrophy were also common, and a small number of patients with encephalitis also showed abnormal contrast enhanced lesions. Of the 26 patients who underwent lumbar puncture, elevated protein concentrations in the CSF were found in 21 cases. The *NOTCH2NLC* gene testing in 53 patients showed GGC repeat number of 102.98 ± 23.52. Among all diagnosed patients, 80–99 and 100–119 were the most common GGC repeat number intervals for NIID (Figure 5A). For patients with NIID presented with cognitive impairment, tremor, bladder dysfunction, disturbance of consciousness, and sensory disturbance, 100–119 was the most common GGC repeat mutations interval, especially for consciousness impairment, which was significantly higher than the other intervals (Figures 5B–F). For patients presenting with muscle weakness and headache, 80-99 was the most common GGC repeat mutations interval (Figures 5G,H). Among the patients with the seven main clinical manifestations mentioned above, the number of GGC repeat expansions was significantly higher in patients with muscle weakness than in other manifestations, patients with bladder disfunction had the lowest number of GGC repeats (Figure 6).

**TABLE 1:** Summary of clinical features of NIID patients.

	Patient 1	Patient 2	All 63 patients
Sex ratio (male/female)	Male	Female	27/34 (2NA)
Age of onset (years)	53	56	54.12 ± 14.12 (*n* = 63)
Age of diagnosis (years)	63	62	59.08 ± 13.99 (*n* = 59)
Disease duration (years)	10	6	7.7 ± 10.12 (*n* = 59)
**Clinical manifestations**			
Main initial symptom	Tremor	Tremor	Tremor (13/54); Cognitive impairment (13/54)
Cognitive impairment	Y	Y	45/56 (80.36%)
Headache	N	Y	21/40 (52.5%)
Dizziness	N	N	9/27 (33.33%)
Vision disorder	Y	N	7/31 (22.58%)
Ataxia	N	N	14/49 (28.57%)
Parkinsonism			
Tremor	Y	Y	30/47 (63.83%)
Rigidity	N	N	3/33 (9.1%)
Bradykinesia	N	N	NA
Autonomic dysfunction			
Vomiting	N	N	9/35 (25.71%)
Bladder dysfunction	N	N	22/47 (46.81%)
Syncope	N	N	6/12 (50%)
Miosis	N	N	14/48 (29.17%)
Peripheral neuropathy			
Muscle weakness	Y	N	26/56 (46.43%)
Sensory disturbance	N	N	14/50 (28.00%)
**Neurological attack**			
Disturbance of consciousness	N	N	21/37 (56.76%)
Stroke-like episode	Y	N	5/12 (41.67%)
**Executive function test**			
MMSE	NP	25	20.54 ± 8.85 (*n* = 26)
MoCA	10	13	17.19 ± 6.45 (*n* = 16)
FAB	NP	NP	10.67 ± 4.42 (*n* = 6)
Abnormal EMG	Y	N	24/28 (85.71%)
Abnormal EEG	N	Y	6/10 (60%)
**Brain MRI**			
DWI U-fiber high signal	Y	Y	51/63 (80.96%)
Severe leukoencephalopathy	Y	Y	29/35 (82.86%)
Ventricular distension	Y	Y	23/38 (60.53%)
**Laboratory data**			
Serum CK (male:38∼174 U/L; female:26∼140 U/L)	101.8	33.2	NA
HgbA1c (4.2∼6.4%)	5.8	5.4	NA
CSF			
Cell (× 10^6^/L)	1	16	NA
Protein (0–500 mg/L)	254	473	21/29 (72.41%) elevated
Glucose (2.5–4.5 mml/L)	3.4	3.4	NA
Positive skin biopsy	Y	Y	63/63 (100%)
GGC repeats	148	131	102.98 ± 23.52 (*n* = 53)

Y, yes; N, no; NA, not access; MMSE, mini-mental state examination; MoCA, montreal cognitive assessment; FAB, frontal assessment battery; EMG, electromyography; EEG, electroencephalogram; CSF, cerebrospinal fluid.

## Discussion

In this study, we report two classical NIID patients diagnosed genetically and pathologically, and we also reviewed all NIID patients diagnosed with the same criteria, and summarized the clinical features of 63 patients with NIID. We found that patients with NIID diagnosed with the abnormal *NOTCH2NLC* GGC repeats and skin biopsy results were mainly from East Asian countries, with slightly more females than males, and almost all patients developed the disease in adulthood, especially in the elderly. Cognitive impairment and tremor are not only the most common symptoms of NIID, but also the most common initial symptoms. In addition, patients with NIID may also present with multiple manifestations of neurological impairment such as headache, autonomic dysfunction such as bladder dysfunction, and attack of loss of consciousness. Our study also found that the number of *NOTCH2NLC* GGC repeats was higher in patients presenting with muscle weakness than in those presenting with other clinical symptoms, which may suggest there is a potential relationship between *NOTCH2NLC* GGC repeats and different symptoms in NIID. In our study, 80.96% (51/63) of NIID patients showed abnormal DWI signals, which is related to lesions in the brains of NIID, while there is small percentage of patients with NIID who do not exhibit abnormalities on DWI and are misdiagnosed. The highly diverse clinical presentations of NIID poses difficulties in clinical diagnosis. Combining gene testing and pathological studying for diagnosis can improve the diagnostic reliability of NIID.

Cognitive dysfunction and abnormal subcortical signals were the most common features for NIID in our study, which may be associated with pathological spongiotic changes in the subcortical white matter proximal to the U-fibers (Yokoi et al., 2016). The diverse clinical manifestations of NIID are associated with abnormal GGC repeat expansion of the *NOTCH2NLC* gene leading to intranuclear inclusion body invasion into different tissues and cells of the nervous system and other systems. Since all the nervous system (central, peripheral, and autonomic) may be affected, and the clinical manifestations depend on the site involved, the clinical presentation vary widely. Although NIID has previously been proposed to be classified into different subtypes based on different symptoms (Sone et al., 2016; Tian et al., 2019), we believe that it is difficult to determine what the main symptom of patients is because many NIID patients have overlapping symptoms.

The clinical presentation of NIID is similar to the *NOTCH2NLC*-related GGC repeat expansion disorders (NRED), a concept that refers to all diseases with the GGC repeat expansion in the 5′UTR of *NOTCH2NLC* gene. To date, NRED includes Parkinsonism-related disorders (Tian et al., 2019; Ma et al., 2020), Alzheimer’s disease (Tian et al., 2019), dementia (Tian et al., 2019; Jiao et al., 2020), multiple system atrophy (Fang et al., 2020), essential tremor (Sun et al., 2020), leukoencephalopathy (Okubo et al., 2019), amyotrophic lateral sclerosis (ALS) (Yuan et al., 2020), oculopharyngeal distal myopathy (OPDM) (Ogasawara et al., 2020), peripheral neuropathy and myopathy (Yu et al., 2021; Huang et al., 2022a). It has been noted that in NRED, the muscle weakness-dominant phenotype has the largest GGC repeat size, usually over 200 repeats, the dementia-dominant phenotype usually has a repeat size between 100 and 200 repeats, and patients with the essential tremor-dominant phenotype usually have about 100 repeats, Parkinson’s disease dominant phenotype with less than 100 repeats (Huang et al., 2022b). Similar to the results of the NRED study, we found that the number of GGC repeats in

NIID patients was significantly higher in patients presented with muscle weakness than in those with other manifestations. However, there was no significant correlation between the number of GGC repeats and the other major clinical symptoms except muscle weakness. Patients with NIID in our study rarely showed greater than 160 GGC repetitions, with most between 80 and 119 repetitions. The specific relationship between genotype and phenotype of NIID needs to be validated by clinical trials that include more patients.

*NOTCH2NLC* is one of the three human-specific *NOTCH2NL* related genes (*NOTCH2NLA*, *NOTCH2NLB*, and *NOTCH2NLC*) (Sone et al., 2019), and it is essential for NIID and NRED. The mechanism of GGC repeat amplification in the 5′UTR of *NOTCH2NLC* gene leading to NIID and NRED is still under investigation. It has been shown that the *NOTCH2NL* gene expresses proteins that activate the Notch signaling pathway and extends cortical neurogenesis by delaying the differentiation of neural progenitor cells, and also leads to recurrent neurodevelopmental disorders (Fiddes et al., 2018). Aberrant polyglycine (polyG) protein aggregation may be a bridge linking *NOTCH2NLC* GGC repeat expansion to intranuclear inclusion body formation. It was found that *NOTCH2NLC* polyG induces cytotoxicity and impairs nucleocytoplasmic transport and may be the mechanism causing neuronal cell damage as well as neurodegeneration (Boivin et al., 2021; Zhong et al., 2021).

Few studies have focused on the relationship between NIID and cerebrovascular disease. We found that some NIID patients present with motor and sensory impairment, stroke-like episodes, disturbance of consciousness, and other symptoms that usually occur in patients with cerebrovascular disease, while severe cerebral white matter lesions, ventricular dilatation, and cerebral atrophy are also common imaging changes in cerebral small vessel disease. The results of a recent study demonstrate that intermediate-length and longer-length GGC repeat expansions in *NOTCH2NLC* gene are associated with sporadic cerebral small vessel disease (Wang et al., 2022), suggesting a possible common pathogenic mechanism between abnormal GGC repeat expansions and cerebrovascular disease. Another recent study demonstrated the presence of cerebral microbleeds and intranuclear inclusions in endothelial cells in another neurodegenerative disease, Fragile X-associated tremor/ataxia syndrome (FXTAS), and the underlying pathogenic mechanism of FXTAS compromises the cerebrovascular system (Salcedo-Arellano et al., 2021). Of interest, both NIID and FXTAS are trinucleotide CGG repeat diseases and PolyG-related diseases (Boivin and Charlet-Berguerand, 2022). Whether trinucleotide CGG repeat or PolyG protein aggregation are common pathogenic mechanism in neurodegenerative diseases and cerebrovascular diseases remains to be studied in detail.

Eosinophilic intranuclear inclusion bodies are not only present in neurons and glial cells of NIID patients, but can also be observed in adipocytes, sweat cells, fibroblasts and other cells (Takahashi-Fujigasaki, 2003; Sone et al., 2011). Under electron microscopy, these intranuclear inclusions can be found to consist of fibrils with a diameter of approximately 8–12 nanometers in diameter, and they are also immunopositive for anti-ubiquitin, anti-p62 and anti-SUMO1 (Lu and Hong, 2021). In addition, among the 26 patients who underwent lumbar puncture, elevated protein concentrations in the CSF were found in 21 cases. Elevated protein concentration in the CSF may be one of the laboratory test features of NIID, but it does not occur in everyone and may be related to the different disease courses and neurological involvement of patients.

Symmetrical high signals at the corticomedullary junction on DWI is considered a characteristic imaging presentation in patients with NIID and has been widely used to screen patients with NIID in the clinic since it was proposed by sone in 2014 (Sone et al., 2014). Skin biopsy and genetic testing in patients with the above MRI findings has higher yield for diagnosis. It should be noted that not all NIID patients present with symmetric high signals at the corticomedullary junction in DWI, and those who do present do not always present the whole course of the disease. Severe leukoencephalopathy, ventricular distension, and cerebral atrophy are also common MRI imaging findings in patients with NIID. In addition, patients with NIID presenting as encephalitis may also have reversible cortical swelling and abnormal enhancing lesions on MRI (Ni et al., 2022).

It is important to note that both skin biopsy and *NOTCH2NLC* genetic testing are necessary tests for the diagnosis of NIID. Although the non-invasive nature of genetic testing makes it easier to perform, a positive genetic test alone does not fully confirm the diagnosis of NIID, as there may be NERD and asymptomatic gene positivity present (Deng et al., 2022). Only positive pathology results are also not sufficient to diagnose NIID, multiple neurodegenerative diseases, neurogenetic diseases, viral infectious diseases and myopathies can also appear intranuclear inclusion bodies (Lu and Hong, 2021). Therefore, the above two tests are complementary tests to confirm the diagnosis in patients with NIID.

Fragile X-associated tremor/ataxia syndrome is a disease caused by a CGG trinucleotide repeat amplification in the 5′UTR of the *FMR1* gene, which is easily misdiagnosed as NIID. In addition to clinical symptoms similar to NIID, FXTAS may also reveal abnormally high signal at the corticomedullary junction in DWI (Toko et al., 2021) and the presence of eosinophilic intranuclear inclusions in hippocampal neurons and glia (Gelpi et al., 2017). Essential tremor, Parkinson’s disease and other diseases that can cause NRED also need to be differentiated from NIID. Therefore, both positive genetic and pathological findings for NIID are important for differentiation from other diseases.

In conclusion, NIID is a neurodegenerative disease caused by abnormal trinucleotides repeat and aberrant polyG protein aggregation, and easily misdiagnosed as FXTAS and NRED. NIID mostly occurs in the elderly population in East Asia, with cognitive dysfunction as the most common symptom. Staging NIID based on clinical presentation is inappropriate because most patients with NIID have overlapping symptoms. Similar to NRED, patients presenting with muscle weakness in NIID have a higher number of GGC repeats. However, there was no significant association between the number of different GGC repetitions and different symptoms other than muscle weakness. Although the high linear intensity of the corticomedullary junction in DWI cannot be used to diagnose NIID, it is effective in helping to confirm the diagnosis in patients with suspected NIID. A positive genetic and pathological result is the criterion for the diagnosis of NIID. Abnormal trinucleotides repeat and PolyG protein aggregation maybe common pathogenic mechanism in neurodegenerative diseases and cerebrovascular diseases, which needs to be confirmed by more studies.

## Data availability statement

The original contributions presented in this study are included in the article/supplementary material, further inquiries can be directed to the corresponding author.

## Ethics statement

The studies involving human participants were reviewed and approved by the Sixth Medical Center of Chinese PLA General Hospital. The patients/participants provided their written informed consent to participate in this study. Written informed consent was obtained from the individual(s) for the publication of any potentially identifiable images or data included in this article.

## Author contributions

BZ designed and wrote the manuscript. MY and QY contributed to the conception and carried out a logic examination of the manuscript. ZW, YZ, and SP participated in the review of medical professional knowledge of the manuscript. MY, QY, and BZ helped with proofreading and revision. YY and XQ contributed to the conception and critically revised the manuscript. All authors contributed to the article and approved the final version.
